# A functional *tonB* gene is required for both virulence and competitive fitness in a chinchilla model of *Haemophilus influenzae* otitis media

**DOI:** 10.1186/1756-0500-5-327

**Published:** 2012-06-25

**Authors:** Daniel J Morton, Randy J Hempel, Thomas W Seale, Paul W Whitby, Terrence L Stull

**Affiliations:** 1 Department of Pediatrics, University of Oklahoma Health Sciences Center, Oklahoma City, Oklahoma, 73104, USA; 2 Department of Microbiology and Immunology, University of Oklahoma Health Sciences Center, Oklahoma City, Oklahoma, 73104, USA

## Abstract

****Background**:**

*Haemophilus influenzae* requires heme for aerobic growth and possesses multiple mechanisms to obtain this essential nutrient.

****Methods**:**

An insertional mutation in *tonB* was constructed and the impact of the mutation on virulence and fitness in a chinchilla model of otitis media was determined. The *tonB* insertion mutant strain was significantly impacted in both virulence and fitness as compared to the wildtype strain in this model.

****Conclusions**:**

The *tonB* gene of *H. influenzae* is required for the establishment and maintenance of middle ear infection in this chinchilla model of bacterial disease.

## **Background**

*Haemophilus influenzae* is a fastidious facultatively anaerobic Gram-negative bacterium that causes a range of human infections including otitis media, meningitis, epiglottitis and pneumonia [1,2]. *H. influenzae* lacks all enzymes in the biosynthetic pathway for the porphyrin ring, and as a result is unable to synthesize protoporphyrin IX (PPIX), the immediate precursor of heme. Since *H. influenzae* cannot synthesize PPIX the organism has an absolute growth requirement for an exogenous source of either heme or PPIX in the presence of iron [3,4]. As a result of this growth requirement, *H. influenzae* has evolved a complex multifunctional array of uptake mechanisms to ensure that it is able to utilize available porphyrin or iron *in vivo*[5]. These mechanisms include numerous outer membrane proteins (OMPs) that bind one or more host heme- and iron- containing proteins including hemoglobin, hemoglobin-haptoglobin, heme-hemopexin and heme-human serum albumin complexes, and ferritransferrin [5-9]. All of these *H. influenzae* OMPs that bind host heme- or iron- containing proteins are TonB-dependent transporters (TBDT) [5]. The outer membrane of Gram-negative bacteria hinders the uptake of essential nutrients, and, while small molecules passively diffuse through porins, substrates that are too large to pass through the porins and/or are present at very low concentrations require energized transport [10,11]. A cytoplasmic transmembrane protein complex composed of three proteins, TonB, ExbB and ExbD, spans the periplasm and interacts with specific TBDTs. This TonB complex transduces the proton motive force of the cytoplasmic membrane to energize transport of substrates through a specific TBDT [10]. Mutations of many of the TBDTs of *H. influenzae* have been shown to have significant impacts on virulence in animal models of infection, for example complete deletion of the complement of hemoglobin-haptoglobin binding proteins (Hgps) in a nontypeable strain significantly reduces the severity of middle ear infection in the chinchilla model of otitis media [12]. Deletion of the complement of *hgps* in a type b strain had a significant impact on virulence in a weanling rat model of bacteremia but not in a 5-day old infant rat model of bacteremia [13]. Similarly, mutation of the heme-hemopexin acquisition protein HxuC impacted virulence in the weanling but not in the 5-day old infant rat [13]. However, a mutant strain lacking both the Hgps and HxuC was unable to sustain bacteremia in 5-day old rats [13]. Mutation of an additional TBDT designated Hup (heme utilization protein) had no impact on virulence in rat models of disease [7]. In addition to the TBDTs many other proteins have been shown to be involved in heme acquisition and in virulence in rat models of invasive disease, including the periplasmic heme-binding protein HbpA, lipoprotein *e*(P4) and the tellurite resistance protein TehB [14-16]. These data highlight the complexity of the heme acquisition systems of *H. influenzae* and their potential roles in virulence.

The *tonB* gene of *H. influenzae* has previously been mutated and shown to be essential for the utilization of transferrin bound iron, hemoglobin, hemoglobin-haptoglobin, heme-hemopexin and low levels of heme [17,18]. In addition a *tonB* mutant of the type b *H.influenzae* strain Eagan was avirulent in an infant rat model of bacteremia [17].

The goal of the present study was to determine if the TonB system is essential for the establishment and maintenance of otitis media in the chinchilla, a widely used model of human otitis media.

## **Methods**

### ***Bacterial strains and growth conditions***

Nontypeable *H. influenzae* strain 86-028NP is a minimally passaged nasopharyngeal isolate from a pediatric patient who underwent tympanostomy and tube insertion for chronic otitis media at Columbus Children’s Hospital. Strain 86-028NP has been extensively characterized in chinchilla models of otitis media [19-21]. *H. influenzae* was maintained long-term in 10% skim milk at −80°C. *H. influenzae* was routinely grown on chocolate agar with bacitracin (BBL, Becton-Dickinson, Sparks, MD, USA) at 37°C. When necessary, *H. influenzae* was grown on brain heart infusion (BHI) agar (Difco, Becton-Dickinson, Sparks, MD, USA) supplemented with 10 μg ml^-1^ heme and 10 μg ml^-1^ β-NAD (supplemented BHI; sBHI) and the appropriate antibiotic(s). Heme-deplete growth was performed in BHI broth supplemented with 10 μg ml^-1^ β-NAD alone (heme-deplete BHI; hdBHI).

### ***Heme sources***

Human serum albumin (HSA), and heme (as hemin) were purchased from Sigma. Stock heme solutions (1 mg ml^-1^ heme in 4% v/v triethanolamine) were prepared as previously described [22]. Heme-albumin complexes were prepared as previously described [7].

### ***Construction of a*tonB*insertional mutant***

The genome of strain 86-028NP has been fully sequenced [23], and the *exbBexbD* and *tonB* genes in this strain are designated with the locus numbers NTHI0358, NTHI0359 and NTHI0360 respectively. An insertional mutation in *tonB* (NTHI0360) was constructed as follows. A pair of primers was designed for use in the PCR, based on the available *H. influenzae* genomic sequence, to amplify an ~1930-bp region encompassing the entire *tonB* gene. Primers were designated TONB-1 and TONB-2 and had the respective sequences 5’-AATGGCAAGATCAAAACGG-3’ and 5’-CCTTATGTTGGATTACTTGG-3’. PCRs were performed in a 50 μl volume using 100 ng of *H*. *influenzae* chromosomal DNA as template, and the reactions contained 2 mM MgCl_2_, 200 μM each dNTP and 2 U of FastStart Taq DNA Polymerase (Roche, Indianapolis, IN, USA). The PCR was carried out for 30 cycles with each cycle consisting of denaturation at 95°C for 1 min, annealing for 1 min at 52°C and primer extension at 72°C for 2 min with one final extension for 30 min. A PCR product of the expected size was obtained and successfully cloned into the TA cloning vector pCR2.1-TOPO (Invitrogen); a plasmid harbouring the correct insert was confirmed by automated sequencing and designated pDJM15. pDJM15 was linearized at a unique *Ssp*I restriction site within the coding sequence of *tonB*. The zeocin resistance marker was excised from pEM7/Zeo (Invitrogen) by digestion with *Eco*RV and *Pvu*II and the ~600-bp fragment containing the zeocin marker was ligated to linearized pDJM15 to yield pDJM19. *H. influenzae* strain 86-028NP was transformed to zeocin resistance using pDJM19 by the static aerobic method as previously described with selection on zeocin at 25 μg ml ^-1^[7,12]. A zeocin resistant 86-028NP transformant was confirmed as correct by sizing and sequencing of a PCR product and was designated HI2280.

### ***Growth studies***

Growth studies were performed using the Bioscreen C Microbiology Reader (Oy Growth Curves AB Ltd., Helsinki, Finland) as previously described [24,25].

### ***Chinchilla models of otitis media***

A total of 15 adult chinchillas (*Chinchilla lanigera*) with no evidence of middle ear infection by either otoscopy or tympanometry at the beginning of the study were used. Animals were rested for at least 7 days upon arrival to acclimate them to the vivarium. After acclimation, chinchillas were challenged with *H. influenzae* in two separate experiments.

In the first experiment groups of five chinchillas were challenged in both ears transbullarly with approximately 2000 c.f.u. of either NTHi strain 86-028NP or its *tonB* mutant derivative HI2280 in order to compare virulence of the two strains. Transbullar inocula were delivered in 300 μl 0.1% gelatin in PBS by direct injection of bacterial suspensions into the superior bullae. Actual challenge dosages received were confirmed by plate count. On days 4, 7, 11, 14 and 17 days post challenge middle ear effusions (MEE) were collected by epitympanic tap, i.e. withdrawl of fluids from the middle ear cavity using a 1.5 inch 25-gauge hypodermic needle [26]. On epitympanic tap the minimum amount of fluid required to perform a dilution series and plating to determine bacterial titers was withdrawn. Ears were scored as “dry” (i.e. no detectable MEE) when an ear was successfully tapped and no evidence of effusion was seen when the plunger of a 1 ml syringe was pulled back maximally. In some cases ears were scored as “ISVP” (insufficient volume to plate) when there was any evidence of effusion, which in some cases manifested as bubbles in the hub of the syringe, but the volume was insufficient to perform a dilution series. Although bacterial titers could not be determined for ISVP ears such ears were considered positive for the presence of MEE.

Bacterial titers were determined using a modification of the track-dilution method of Jett et al as previously described [7,27]. Serial dilutions (0 to 10^-5^) of freshly recovered MEE were made in 0.1% gelatin in PBS and 10 μl aliquots from each dilution were plated in triplicate on sBHI and all plates were incubated at 37°C for 48 hours to quantify c.f.u. NTHi ml ^-1^. Since heme is not required for anaerobic growth plates were incubated both aerobically and anaerobically to remove any potential impact of the *tonB* mutation on detection of bacteria.

In the second experiment a group of 5 chinchillas was challenged transbullarly with an inoculum containing equal numbers of NTHi strain 86-028NP and its *tonB* mutant derivative HI2280 (total of approximately 2500 c.f.u.) to quantify differential fitness of the two strains. All ears were tapped for collection of MEE on days 4, 7, 11, 15 and 18 days post infection. Each recovered MEE was plated on sBHI and on sBHI containing 7.5 μg ml ^-1^ zeocin to determine total bacterial titer and the titer of the mutant strain respectively (7.5 μg ml ^-1^ zeocin was used in these experiments since it is the lowest concentration we have found to be adequate for differentiation of zeocin-sensitive and zeocin-resistant strains). The competitive index (CI) was calculated for all ears at all time-points in experiment 2 and is defined as the ratio of the output mutant/wildtype ration to the input mutant/wildtype ratio.

Animal procedures have been described in detail elsewhere [19,26,28]. The protocol for use of animals in this study was reviewed and approved by the Institutional Animal Care and Use Committee of the University of Oklahoma Health Sciences Center.

### ***Statistics***

Statistical comparisons of growth between strains under the same growth conditions *in vitro* were made using the Mann–Whitney test. Percentages of infected ears yielding a detectable effusion and percentages of effusions with detectable wildtype or mutant bacteria were compared using Fisher’s Exact test.

Analyses were performed using Analyse-It for Microsoft Excel v2.22 (Analyze-It Software Inc., Leeds, England). A *P* value < 0.05 was taken as statistically significant.

## **Results and discussion**

**Figure 1:**
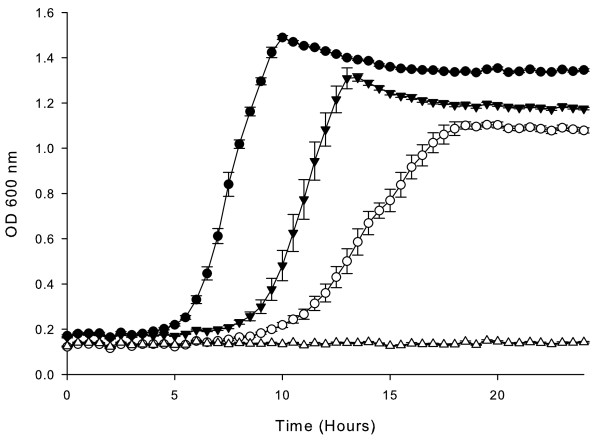
**- Growth of*H. influenzae*strains with either free heme or the heme-hemun serum albumin complex as the sole heme source.** Growth of the nontypeable *H. influenzae* strain 86-028NP and the *tonb* insertion mutant strain HI2280 in hdBHI supplemented with heme or heme-human serum albumin as the sole heme source. Wildtype strain 86-028NP with heme at 10 μg ml^-1^ (solid circles) and with heme-human serum albumin at 50 ng ml^-1^ heme equivalent (open circles). The *tonB* mutant strain HI2280 with heme at 10 μg ml^-1^ (solid triangles) and with heme-human serum albumin at 50 ng ml^-1^ heme equivalent (open triangles). Results are mean ± SD for quintuplicate results from representative experiments. Using the Mann Whitney test to compare growth of 86-028NP and HI2280 over the entire 24 hour growth period in either heme source *P* < 0.0001.

An insertional mutation in the *tonB* gene of *H. influenzae* strain 86-028NP was constructed as described in the Methods section. The *tonB* mutant strain (HI2280) contains a zeocin resistance marker disrupting codon 147 of the *tonB* gene. To confirm the anticipated phenotype, the *tonB* mutant was compared to the wildtype strain in growth curve analyses for the ability to utilize various porphyrin sources. The mutant strain grew significantly less well than the wildtype strain in heme at 10 μg ml^-1^ and did not grow at all in heme-HSA at 50 ng ml^-1^ heme equivalent (Figure 1). In addition the *tonB* mutation abrogated the ability to utilize either heme at lower concentrations or hemoglobin-haptoglobin complexes as the sole heme source (data not shown). These observations with respect to the impact of a *tonB* mutation on heme source utilization are further supported by the data of Jarosik *et al*[17,18], who demonstrated in two type b strains and one nontypeable strain that mutation of *tonB* abrogated the ability to utilize various heme sources including heme, hemoglobin-haptoglobin and heme-hemopexin. Jarosik et al also showed that complementation of the *tonB* mutation with the complete *exbB exdD tonB* operon corrected the in vitro phenotypes. However, repeated attempts to complement the mutation in strain 86-028NP using various strategies have been unsuccessful to date. We initially attempted to clone the entire *exbBexbDtonB* operon, in order to drive production from the native promoter, in *E. coli* in both high-copy and low-copy number plasmids; cloning in *E. coli* failed presumably due to toxicity. Subsequently we attempted to clone the operon directly into *H. influenzae* using either the shuttle vector pACYC184 or the suicide vector pASK5 [29]. Although these attempts were not successful, we would predict that such a complement would correct the phenotypes reported herein similarly to the previous reports.

In addition to the impact of *tonB* mutation on heme source utilization, we and others have previously shown that utilization of many of these heme sources is abrogated by mutations in specific TBDTs [6-8,30]. For example utilization of heme-HSA is dependent on a functional HxuC protein [8], and the utilization of hemoglobin-haptoglobin complexes requires the presence of a functional hemoglobin-haptoglobin binding protein (HgpA, HgpB, or HgpC) [6,25].

Having established that the *tonB* mutant strain exhibited the expected in vitro phenotype, we compared the mutant and wildtype strains for their respective abilities to establish and maintain infection of the middle ear in chinchilla models of otitis media.

The TonB protein has previously been shown to be essential for virulence *of H. influenzae* type b strains in the infant rat model of bacteremia [17]. However, there have been no reports of the impact of *tonB* mutations on virulence in other clinically relevant models of *H. influenzae* disease, including models of otitis media. Two separate experiments were performed, one to assess the impact of the *tonB* mutation on virulence by comparison of two groups of chinchillas infected with individual strains, and the second to assess the impact of the mutation on competitive fitness by infection of a cohort of animals with equal numbers of both strains.

**Figure 2:**
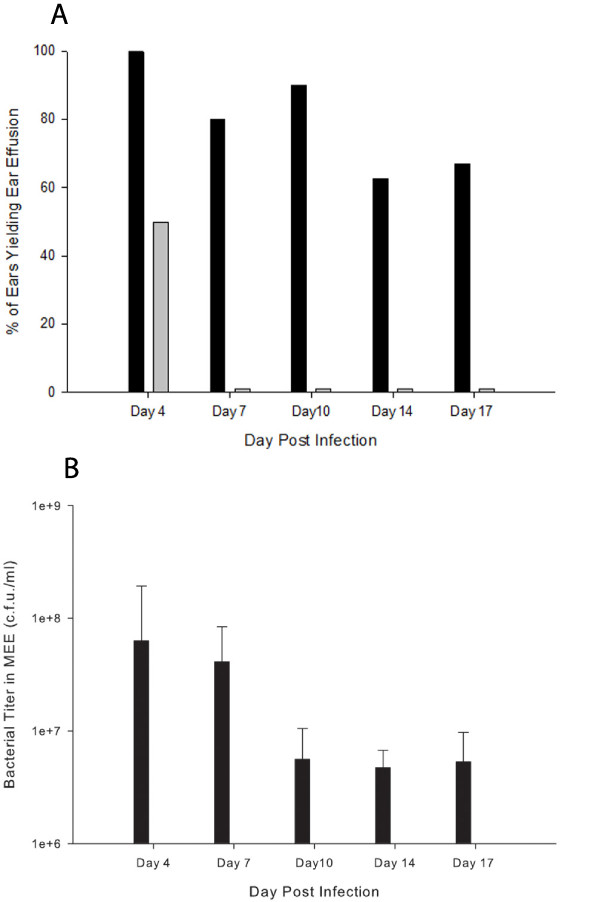
**– Percentage of infected ears yielding a middle ear effusion and bacterial titers in middle ear effusions.A)** Percentage of successfully tapped ears infected with either the wildtype strain 86-028NP (black columns) or the *tonB* mutant strain HI2280 (grey columns) yielding a middle ear effusion. Using Fisher’s Exact test to compare percentages of ears infected with either strain yielding effusions on given days: on day 4 *P* = 0.0325, on day 7 *P* = 0.0007, on day 10 *P* = 0.0004, on day 14 *P* = 0.0256 and on day 17 *P* = 0.015. **B)** Bacterial titers in middle ear effusions from ears infected with the wildtype strain 86-028NP (black columns). No bacteria were detected in middle ear effusions recovered on day 4 from ears infected with the *tonB* mutant strain HI2280. On days 7 through 17 no middle ear effusions were recovered from ears infected with the *tonB* mutant strain HI2280.

In our virulence study experiment, two groups of five chinchillas were transbullarly infected bilaterally with either the wildtype strain 86-028NP or the isogenic *tonB* mutant HI2280 (actual infective doses were 1,880 c.f.u. for 86-028NP and 1,150 c.f.u. for HI2280). On day 4 post challenge 9 of 9 successfully tapped ears challenged with the wildtype strain yielded effusions. When animals were challenged with the *tonB* mutant a different result occurred. Five of 10 successfully tapped ears were dry in the *tonB* mutant challenged cohort (Figure 2A). All MEE recovered from the wildtype infected ears on day 4 yielded bacterial titers in the range 5 x 10^6^ to 4 x 10^8^ c.f.u. ml ^-1^ (average titer 6.3 x 10^7^ ± 1.3 x 10^8^ c.f.u. ml ^-1^). In contrast the five MEEs recovered from mutant-challenged ears on day 4 yielded no colonies on culture under either aerobic or anaerobic conditions (Figure 2B). Anaerobic conditions were used in addition to aerobic since *H. influenzae* does not require heme for anaerobic growth [5] and on no occasion was there any difference in titers determined in the two growth conditions. On days 7, 10, 14 and 17 post-infection, all of the successfully tapped ears challenged with the *tonB* mutant were dry. In contrast, in ears challenged with the wildtype strain the majority yielded a MEE over the same time period, with average bacterial titers consistently in the 10^6^ to 10^8^ range (Figure 2). In addition, on day 17 five of the mutant challenged ears were lavaged with 200 μl of 0.1 % gelatin in PBS. None of the five lavage fluids from ears infected with the mutant strain yielded detectable bacteria on either aerobic or anaerobic culture. On day 17 all animals were killed and the bullae were excised from selected animals. Bullae were excised from four ears challenged with the wildtype strain as well as from all unlavaged ears challenged with the *tonB* mutant strain. On macroscopic examination of the four bullae excised from wildtype infected ears all contained dense opaque material that has been termed biofilm and is typically found in *H. influenzae* infected ears at 7 days post-infection and beyond [31,32]. In contrast, on macroscopic examination of the five bullae excised from the mutant infected animals none contained similar material (data not shown).

**Figure 3:**
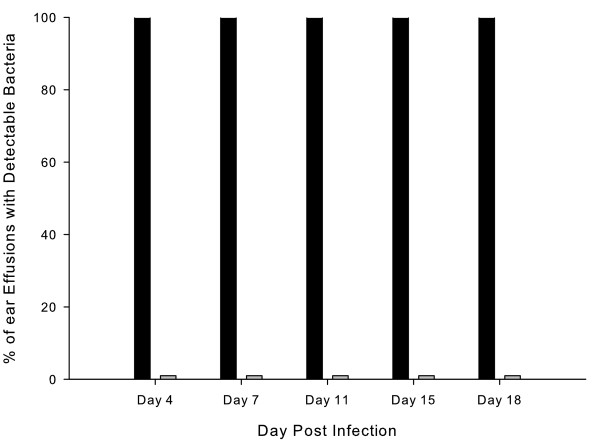
**– Percentage of middle ear effusions with detectable wildtype or mutant bacteria.** Percentage of successfully tapped ears infected with a mixture of equal numbers of the wildtype strain 86-028NP and the *tonB* mutant strain HI2280 containing detectable colonies of the wild type strain (black columns) or the mutant strain (grey columns). Using Fisher’s Exact test to compare percentages of effusions with detectable wildtype or mutant strains *P* < 0.008 on all days.

A second experiment was designed to assess competitive fitness of the wildtype and *tonB* mutant when the two strains were used to co-challenge chinchilla ears. A group of 5 chinchillas was infected transbullarly with equal numbers of 86-028NP and *tonB* mutant cells (actual infective dose 4330 c.f.u equally divided between the two strains). On days 7, 11 and 15 all ears were positive for the presence of MEE while on days 4 and 18 respectively 90 % and 75 % had MEE. In all MEE only the wildtype strain was detected with average titers ranging from 2.1 x 10^8^ ±2.1 x 10^8^ to 2.6 x 10^6^ ±2.4 x 10^7^; no bacteria were detected on media containing zeocin under either aerobic or anaerobic incubation (Figure 3). These data allow quantification of differential fitness between strains by calculation of CI. CI is defined as the mutant/wildtype ratio in the output sample divided by the same ratio in the inoculum . The CI for all ears at all time-points was 0; a CI of 1 would indicate that the mutant strain is able to grow as well as the wildtype strain and a CI < 1 indicates that mutant growth is attenuated. Thus, in this case the mutant is significantly attenuated in comparison to the wildtype strain. Using Fisher’s Exact test to compare percentages of effusions with detectable wildtype or mutant strains on day 4 *P* < 0.0001, on day 7 *P* = 0.0006, on day 10 *P* = 0.0079, on day 14 *P* = 0.0002 and on day 17 *P* = 0.0022. *P* values vary since ears which yielded no effusion or insufficient volume to analyze are excluded from the analysis, thus sample numbers vary by day. Thus, under identical in vivo conditions the wildtype strain markedly out competed its isogenic *tonB* mutant.

## Conclusions

These data demonstrate that expression of TonB is essential for both virulence and competitive fitness of a nontypeable *H. influenzae* strain in a chinchilla model of otitis media. It has previously been shown that a *tonB* mutant of a type b strain is avirulent in the infant-rat model of invasive disease. Thus TonB is essential for establishment of *H. influenzae* disease in multiple clinically relevant animal models of *H. influenzae* disease. Since TonB is required for the function of TBDTs and all of the *H. influenzae* TBDTs appear to be involved in acquisition of heme these data indicate that heme acquisition is an essential process during infection caused by *H. influenzae*. This observation is further supported by the reports of the impact of mutations in specific *H. influenzae* TBDTs, including the hemoglobin-haptoglobin binding proteins (Hgps) and the heme-hemopexin acquisition protein (HxuC), on virulence in animal models [12,13].

In conclusion expression of TonB is required for the establishment and maintenance of infection in an animal model of *H. influenzae* otitis media.

## **Competing interests**

The authors declare that they have no competing interests.

## **Authors' contributions**

All authors contributed to the design and execution of the experiments detailed. DJM constructed the *tonB* mutant strain. DJM and RJH performed animal experiments. DJM drafted the manuscript. PWW, TWS and TLS revised the manuscript. All authors read and approved the final manuscript.
